# PD51 - Children with asthma exposed to Environmental Tobacco Smoke (ETS) and inhaled corticosteroids treatment

**DOI:** 10.1186/2045-7022-4-S1-P51

**Published:** 2014-02-28

**Authors:** Snezana Radic, Zorica Zivkovic, Branislav Gvozdenovic, Sofija Cerovic, Olivera Calovic, Tamara Krivokapic, Olivera Vlahovic, Ksenija Jevtic, Vera Aleksic

**Affiliations:** 1 University Hospital Dr Dragisa Misovic Dedinje, Belgrade, Serbia; 2 PPD Belgrade, Belgrade, Serbia

## Background

Corticosteroids are the most effective anti-inflammatory therapy for asthma. A reduction in histone deacetylase (HDAC) activity is suggested to prevent the anti-inflammatory action of inhaled corticosteroids (IC). Cigarette smoke is known to reduce HDAC expression.

## Aim

To compare the lung function test parameters and the response to the IC in the asthmatic children exposed and not exposed to environmental tobacco smoke (ETS).

## Methodology

527 children (6-16 years) with moderate to severe asthma performed spirometry before and after the 6 months of IC. According to questionnaire, we divided children into two groups: ETS exposed (ETSE, N 337) and ETS free (ETSF, N 190).

## Results

There were 59.9% of boys and 50.1% of girls (mean age 10.83). Average dose of Fluticasone propionate (FP) was 225.11±119.98mcg per day per child. Among ETSE children, 208 were with one, 129 with both smoking parents, 228 had smoking mother and 238 had smoking father. ETSE children received statistically higher dose of FP, and dose of FP incresed with increasing of number of smokers in the family (F=45.41, p<0.001). ETSE children had lower lung function parameters before and after the IC, and the influence of mother and both smoking parents on lung function was more pronounced than fathers alone. After the 6 months of IC, both groups of children significantly improved lung function tests, no difference between groups.

## Conclusion

ETS impaired the lung function growing rate in exposed children with asthma. ETSE children did not have resistance to IC because their airways still response to inhaled corticosteroids. It is necessary to educate smoking parents to protect asthmatic children from tobacco smoke negative influence.

